# Atropine: A Cure for Persistent Post Laparoscopic Pyloromyotomy Emesis?

**DOI:** 10.21699/jns.v6i1.485

**Published:** 2017-01-01

**Authors:** Robert Frank Cubas, Shannon Longshore, Samuel Rodriguez, Edward Tagge, Joanne Baerg, Donald Moores

**Affiliations:** 1 Department of Surgery, Loma Linda University Medical Center, Loma Linda, CA, USA; 2 Division of Pediatric Surgery, Loma Linda University Children’s Hospital, Loma Linda, CA, USA

**Keywords:** Pyloric stenosis, Atropine, Post-pyloromyotomy emesis

## Abstract

Background: Atropine has been used as a successful primary medical treatment for hypertrophic pyloric stenosis. Several authors have reported a higher rate of incomplete pyloromyotomy with the laparoscopic approach compared to open. In this study, we evaluated the use of atropine as a medical treatment for infants with emesis persisting greater than 48 hours after a laparoscopic pyloromyotomy.

Materials and Methods: We performed a retrospective chart review of infants receiving a laparoscopic pyloromyotomy between November 1998 and November 2012. Infants with emesis that persisted beyond 48 hours postoperatively were given 0.01mg/kg of oral atropine 10 minutes prior to feeding. Infants remained inpatient until they tolerated two consecutive feedings without emesis.

Results: 965 patients underwent laparoscopic pyloromyotomy; 816 (84.6%) male and 149 (15.4%) female. Twenty-four (2.5%) received oral atropine. The mean length of stay for patients who received atropine was 5.6 ± 2.6 days, an average of 3 additional days. They were discharged home with a one-month supply of oral atropine. Follow up evaluation did not reveal any complications from receiving atropine. The median follow up was 21 days. None returned to the operating room for incomplete pyloromyotomy. There were 17 (1.8%) operative complications in our series; 9 mucosal perforations, 2 duodenal perforations, and 6 conversions to open for equipment failure or poor exposure. There were 4 (0.4%) post-operative complications: 2 episodes of apnea requiring reintubation and 2 incisional hernias that required a second operation. There were no deaths.

Conclusion: Oral atropine is a viable treatment for persistent emesis after a pyloromyotomy and reduces the need for a second operation due to incomplete pyloromyotomy.

## INTRODUCTION

Laparoscopic pyloromyotomy was first described in 1991 by Alain et al and has become the most widespread approach in the last few years. Initially, the laparoscopic approach was linked with incomplete myotomy and higher complication rates [1-3], all of which has been left as a historical matter as recent studies have demonstrated its equivalence and, in some instances, superiority in terms of complications, operative time and recovery time when compared with the open approach [4]. Nonetheless there is still a 0.3 - 5% rate of incomplete pyloromyotomies with the laparoscopic approach [5, 6].

Although medical therapies, including IV/PO atropine, have been used in the past 50 years, the utility of medical management remains in question and is not currently widely accepted in Europe and the USA as a primary therapy for HPS, as opposed to Japan, where medical management using IV atropine has been reported to be a promising treatment [7, 8].

Atropine is a stereoisomer of hyoscyamine. Significant levels are achieved in the CNS within 30 minutes to 1 hour and the drug disappears rapidly from the blood with a half-life of 2 hours. Its effects on the smooth muscle of the pylorus are accomplished by competitive and reversible binding to M3 receptors, provoking a relaxation of the muscularis propria. It has been used effectively as an antidote for anticholinesterase and muscarine-containing mushroom poisoning, as a preanesthetic medication to inhibit secretions, and during reversal of neuromuscular blockade. In cardiovascular conditions, atropine is used for treatment of symptomatic sinus bradycardia and atrioventricular (AV) nodal block.

At our institution, we have used oral atropine as a rescue therapy for patients with emesis persisting greater than 48 hours after a laparoscopic pyloromyotomy. The aim of our study is to present our experience and outcomes in the management of persistent post-pyloromyotomy emesis in one of the largest population based studies ever presented.

## MATERIALS AND METHODS

After obtaining Institutional Review Board approval, infants undergoing a laparoscopic pyloromyotomy for hypertrophic pyloric stenosis at Loma Linda University Children’s Hospital, between November 1998 and November 2012 were retrospectively reviewed.

Data were collected from electronic medical records and entered on a spreadsheet. The information extracted consisted of demographic data (sex, age, and weight), duration of symptoms, size of the pyloric wall and channel, chloride and bicarbonate serum levels on admission and prior to surgery, intra and post-operative complications, number of post-operative emesis greater than 15mL (measured by weighing the soaked wardrobes or blankets and by estimation of the pediatric nurses), and length of stay.

The diagnosis of HPS was made based on clinical assessment and ultrasonographic findings under standard institutional sonographic criteria (i.e. pyloric muscle thickness of at least 4mm, length of at least 16mm, and lack of peristalsis or passage of fluid through the pylorus).

Infants that underwent an open pyloromyotomy or a concomitant secondary procedure were excluded.

Infants admitted with a HCO3 of 35mEq/L or higher or a Chloride of 85mmol/L or lower were placed in a unit with cardiac and respiratory monitoring. After adequate resuscitation was achieved (indicated by a HCO3 level between 24-27mEq/L and satisfactory urine output), the infant underwent laparoscopic pyloromyotomy.

Infants with emesis that persisted beyond 48 hours postoperatively were given 0.01 mg/kg of oral atropine 10 minutes prior to each feeding. For this purpose we used a 0.1mg/mL solution and dispensed it to the infant by means of a 1mL slip tip syringe. Infants remained in hospital until they tolerated two consecutive feedings of 60 mL without emesis and were sent home with a 30-day provision of oral atropine. Parents were advised to return to clinic for follow up at 7 days after discharge. Some of them needed an additional appointment 1 to 3 weeks after the initial postoperative visit depending on their response or need for further medication.

A two tailed independent t-test was used for comparison of continuous variables. Chi-square and 2-tailed Fisher’s exact probability tests were used for nominal data, p values obtained from these statistical tests were deemed significant if p is less than 0.05.

## RESULTS

Nine hundred and sixty-five patients underwent laparoscopic pyloromyotomy between November 1998 and November 2012, 84.6% were male. The mean age at presentation was 5 weeks ± 2 weeks. The duration of symptoms ranged from 1 to 62 days before evaluation by an urgent care or emergency room physician.

Twenty-four infants (2.5%) developed post-operative emesis for more than 48 hours that required initiation of therapy with oral atropine. On average 20 doses were necessary before the infant could tolerate 2 ounce feeds (60mL). There was a statistically significant difference in the chloremia on admission and length of stay between those that received atropine and those that did not require it. Table1.

No infants returned to the operating room for persistent post-pyloromyotomy emesis. There were 17 (1.8%) operative complications in our series; 9 mucosal perforations, 2 duodenal perforations, and 6 conversions to open for equipment failure or poor exposure. The perforations were all identified at the time of surgery. There were 4 (0.4%) post-operative complications: 2 episodes of apnea requiring reintubation and 2 incisional hernias that required a second operation. No mortalities were recorded. At subsequent clinic appointments, there were no cardiac or respiratory events reported to have occurred, either as an inpatient, or after discharge to home. The median follow up was 21 days (7 to 35 days).

**Figure:**
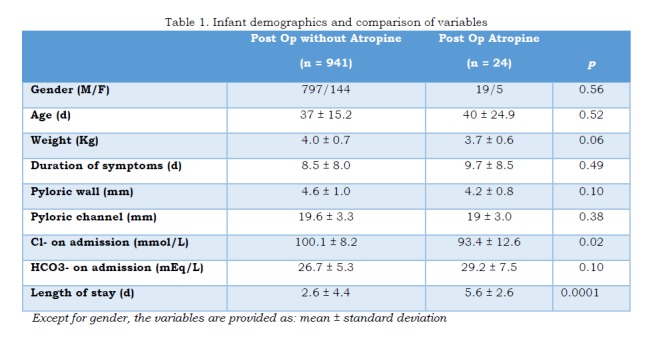
Table 1. Infant demographics and comparison of variables

## DISCUSSION

Postoperative emesis in neonates with HPS after laparoscopic pyloromyotomy is a clinical challenge and requires careful evaluation. It can be a result of incomplete myotomy (4.3%), enteric viral infections (43%), aggressive feeding protocols (40%) or gastro-oesophageal reflux (14%) as established by Castellani [9]. In his study, he re-operated 5 infants with suspected incomplete pyloromyotomy, finding that only 2 had the presumed diagnosis.

In our series, if the diagnosis of persistent post-pyloromyotomy emesis was made, irrespective of the true cause of the vomiting, the treatment was a conservative approach with oral atropine without obtaining further radiologic evaluation due to the known persistence of the characteristic appearance of HPS for several weeks [5].

Over the past 23 years, atropine sulphate has been used increasingly in Japan as primary therapy for HPS. Kawahara reported an average time from initiation of intravenous antimuscarinic therapy to vomiting cessation of 6 days. As atropine sulphate is a cardioactive drug, electrocardiographic and pulse-oximetry monitoring was performed during administration at initiation and dose change. In our series no cardiac or respiratory monitoring was performed (except for severe electrolyte abnormalities), as we used a standard low oral dose. Of note no cardiac events were reported neither in house nor in subsequent clinic follow up. In Kawahara’s series pyloromyotomy was ultimately required in 13% of cases, where vomiting persisted beyond 3 weeks.

In Western countries, medical management of HPS has remained a controversial issue. Meissner [10] evaluated the use of atropine sulphate versus pyloromyotomy and established the failure rate of medical management to be unacceptable (25%) when compared with that of surgical repair (less than 5%). In addition to this, they argued that the increased cost involved in non-operative management was difficult to justify when a relatively immediate surgical option was available with a low complication rate. In contrast, Yamataka [11] found that costs were lower in their medically managed group of patients in Japan, but his numbers were severely criticized by his colleagues in the USA.

## CONCLUSION

Since data on conservative management of HPS with atropine is limited, and because surgery is both safe and effective, the consensus today is that atropine for the treatment of HPS should be used mainly for infants in whom a surgical approach is either not advisable or feasible. However, few studies report its use for the treatment of persistent post-pyloromyotomy emesis. Our study reveals that atropine is safe and effective with negligible side effects, and most importantly, its use allows infants to avoid unnecessary re-operations.

## Footnotes

**Source of Support:** None

**Conflict of Interest:** None
